# Clinical Evaluation of a Novel Combination of Sodium Hypochlorite/Amino Acid and Cross-linked Hyaluronic Acid Adjunctive to Non-surgical Periodontal Treatment: A Case Series

**DOI:** 10.3290/j.ohpd.b4347453

**Published:** 2023-08-30

**Authors:** Egle Ramanauskaite, Vita Machiulskiene, Urte Marija Dvyliene, Meizi Eliezer, Anton Sculean

**Affiliations:** a PhD Student, Clinic of Dental and Oral Pathology, Lithuanian University of Health Sciences, Kaunas, Lithuania. Study design, data acquisition and interpretation, manuscript drafting and revision, gave final approval and agreed to be accountable for all aspects of the work.; b Professor, Clinic of Dental and Oral Pathology, Lithuanian University of Health Sciences, Kaunas, Lithuania. Manuscript drafting and revision, gave final approval and agreed to be accountable for all aspects of the work.; c Periodontist, Clinic of Dental and Oral Pathology, Lithuanian University of Health Sciences, Kaunas, Lithuania. Manuscript revision, gave final approval and agreed to be accountable for all aspects of the work.; d Periodontist, Tel Aviv University, Tel Aviv, Israel. Manuscript drafting and revision, gave final approval and agreed to be accountable for all aspects of the work.; e Professor, Department of Periodontology, University of Bern, Switzerland. Study conception, design and supervision, data interpretation, manuscript drafting and revision.

**Keywords:** cross-linked hyaluronic acid, non-surgical periodontal therapy, periodontitis, sodium hypochlorite/amino acid

## Abstract

**Purpose:**

The adjunctive subgingival application of sodium hypochlorite/amino acid and a mixture of natural and cross-linked hyaluronic acid gels (high molecular weight) has been recently proposed as a novel modality to enhance the outcomes of non-surgical periodontal therapy. The aim of this prospective case series was to evaluate the clinical outcomes obtained following the subgingival application of a combination of sodium hypochlorite/amino acid and a mixture of natural and cross-linked hyaluronic acid (high molecular) gels in conjunction with non-surgical periodontal therapy.

**Material and Methods:**

Twenty-one systemically healthy, non-smoking patients diagnosed with stage II-III, grade A/B periodontitis underwent full-mouth subgingival debridement (SD) performed with ultrasonic and hand instruments. All sites with probing depths (PD) ≥ 4 mm were treated with additional repeated (i.e., 2-3 times) instillation of sodium hypochlorite/amino acid gel in the periodontal pockets prior to and during SRP. Following mechanical debridement, a mixture of natural and cross-linked hyaluronic acid (high molecular) gel was applied in the pockets. The primary outcome variable was PD reduction; changes in clinical attachment level (CAL) and bleeding on probing (BOP) were the secondary outcomes. The clinical parameters were assessed at baseline, 3 and 6 months after therapy.

**Results:**

Compared to baseline, a statistically significant mean reduction of PD values was obtained after 3 and 6 months, amounting to 2.6 ± 0.4 mm, and 2.9 ± 0.4 mm, respectively (p < 0.001). Mean CAL gain measured 2.3 ± 0.5 mm at 3 months and 2.6 ± 0.5 mm at 6 months in comparison to baseline (p < 0.001). Mean reduction of BOP values was 54.9 ± 16.9 % at 3 months and 65.6 ± 16.4 % at 6 months (p < 0.001). The number of moderate pockets (4-5 mm) decreased from 1808 at baseline to 274 at the 6-month evaluation, and the number of deep (≥ 6 mm) pockets dropped from 319 to 3, respectively.

**Conclusion:**

The combination of sodium hypochlorite/amino acid and a mixture of natural and cross-linked hyaluronic acid (high molecular) adjunctive to subgingival debridement may represent a valuable approach to improve the outcomes of non-surgical periodontal treatment.

Periodontitis is a chronic, progressive disease, characterised by expansion of the polymicrobial biofilm at the gingival margin, with the formation of an inflammatory infiltrate that contributes to destruction of connective-tissue attachment to the tooth, alveolar bone resorption and eventually even tooth loss.^1,2,27^

Dental plaque biofilm represents an acquired tissue of bacterial origin that maintains the health of gingival tissues and facilitates interactions between microorganisms and the host.^4,10^ In periodontitis, a disruption of the normal function of the healthy subgingival plaque biofilm is observed, with concomitant disruption of its functional properties, leading to excessive, deregulated inflammation and tissue destruction.^4,13^

Elimination of the biofilm is a key element for the successful treatment of periodontitis. Although thorough subgingival debridement is a cornerstone of periodontal therapy, its effectiveness may be limited by several factors (e.g., deep periodontal pockets, intrabony defects, furcation involvement, operator’s manual skills, the patient’s smoking status, etc.). Therefore, the adjunctive application of antimicrobial chemotherapeutic agents to eliminate or inactivate the periodontal pathogenic microflora at sites where mechanical instrumentation is cumbersome is highly clinically relevant.^22^

It has been suggested that adjunctive aids may enhance the outcomes of mechanical debridement.^22,24,28^ Recently, the novel concept of ‘Clean and Seal’ – based on adjunctive use of two components, i.e., sodium hypochlorite/amino acid (Perisolv, Regedent; Zürich, Switzerland) and a mixture of natural and cross-linked hyaluronic acid (high molecular) (Hyadent BG, Regedent) gels along with mechanical instrumentation – was introduced as an option for non-surgical periodontal therapy.

In fact, preclinical studies have shown that sodium hypochlorite/amino acid gel acts antiseptically in particular against gram-negative species associated with periodontitis and is able to alter biofilm matrices.^3^ Moreover, hyaluronic acid demonstrated bacteriostatic effects on bacterial strains associated with periodontitis and was proven to be beneficial in minimising bacterial contamination of surgical wounds.^11^

Regarding the ‘Clean and Seal’ concept, the cleaning effect is achieved by the activity of the sodium hypochlorite/amino acid gel. Laboratory experiments have demonstrated that sodium hypochlorite/amino acid gel has a softening effect on the extracellular matrix of the biofilm^14^ and therefore, during treatment, both mechanical and chemical reactions act in concert to disrupt the biofilm and remove granulation tissue.^23^ It is noteworthy that this chemomechanical method has no detrimental effect on sound dentin and/or root cementum. The high pH of the product affects calculus and has a softening effect, which makes the cleaning process easier to perform.^23^

The sealing effect is obtained by subsequent application of a mixture of natural and cross-linked hyaluronic acid gel (high molecular). Hyaluronic acid is a major constituent of the extracellular matrix of the skin, joints, eye, and many other tissues and organs.^18^ Numerous in-vitro studies have provided evidence that hyaluronic acid stimulates blood clot formation,^26^ induces angiogenesis^6^ and enhances osteogenesis.^5,15,25^ In addition, hyaluronic acid was found to play a key role in each phase of wound healing by stimulating cell migration, differentiation, and proliferation.^18^

However, at present, clinical data validating the clinical efficacy of the aforementioned treatment concept in patients with untreated periodontitis is lacking. Therefore, the aim of this prospective case series was to evaluate in patients with untreated periodontitis the clinical outcomes obtained with subgingival application of sodium hypochlorite/amino acid and a mixture of natural and cross-linked hyaluronic acid (high molecular) in conjunction with non-surgical periodontal therapy.

## Materials and Methods

### Subject Selection

A total of 21 systemically healthy patients were recruited from new referrals to the Department of Dental and Oral Pathology, Lithuanian University of Health Sciences. The inclusion criteria were: a clinical diagnosis of stage II-III periodontitis,^20^ at least one pocket in each quadrant with pocket depth (PD) ≥ 5 mm; radiographic evidence of bone loss (> 2 mm from cemento-enamel junction [CEJ]); a minimum of 20 teeth (wisdom teeth excluded); no removable prosthesis. The exclusion criteria were: patients already included in other clinical trials; smokers; periodontal treatment during the last 12 months; antibiotic treatment 6 months prior to the start of the trial; antibiotic prophylaxis required for dental treatment; ongoing medication that may affect the clinical features of periodontitis; pregnancy/lactation.

Furthermore, patients were included in the study if they exhibited an adequate level of oral hygiene evidenced by full-mouth plaque score (FMPS) <25%^19^ and full-mouth bleeding score (FMBS) <25%.^16^ Written informed consent was obtained from all patients. Ethical approval was obtained from Kaunas Regional Biomedical Research Ethics Committee (2018-BE-2-87).

### Treatment

Baseline periodontal measurements were obtained two weeks prior to treatment, which was followed by professional supragingival tooth cleaning and individual oral hygiene instructions for all of the included patients. Oral hygiene instructions were reinforced at each follow-up visit, but no further treatment was provided.

**Fig 1:**
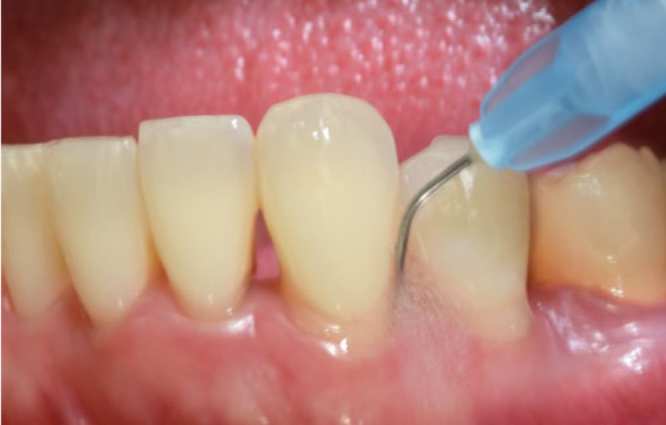
Application of sodium hypochlorite/amino acid gel to the periodontal pocket.

**Fig 2:**
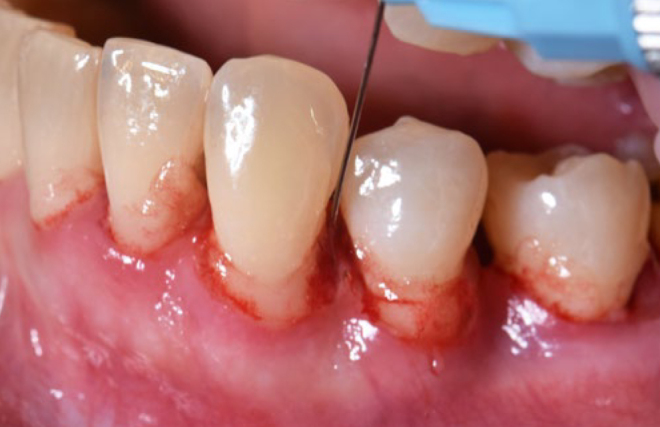
Application of a mixture of natural and cross-linked hyaluronic acid (high molecular) to the periodontal pocket.

Two weeks later, under local anesthesia, all patients underwent full-mouth SD performed with ultrasonic (Satelec/Acteon suprasson newtron ultrasonic scaler; Merignac, France) and hand instruments (LM SharpDiamond 1/2, 7/8, 11/12, 13/14 SD mini Gracey and Gracey curettes). Subsequently, all teeth were polished using a low-abrasive paste (Lunos Super Soft, RDA<5, Dürr Dental; Bietigheim-Bissingen, Germany). Per patient, the average time needed for the treatment was 3 h. All teeth with probing depths (PD) ≥ 4 mm were treated with sodium hypochlorite/amino acid gel (Perisolv, Regedent) injected into the periodontal pockets 60 s prior to and during SD (2-3 times) (Fig 1). No additional rinsing with saline was performed. Mechanical debridement was followed by the subsequent application of a mixture of natural and cross-linked hyaluronic acid (high molecular) gel (Hyadent BG, Regedent) in periodontal pockets measuring ≥ 4 mm (Fig 2).

Periodontal treatment was performed by an experienced periodontist (E.R.).

All patients were advised to follow their regular home oral-hygiene regimen and to refrain using antiseptic mouthwashes during the entire study period.

### Clinical Assessments

The following clinical parameters were assessed using a Williams periodontal probe to the nearest mm (LM 51 ES, LM-Dental; **Parainen**, Finland) at all teeth at six sites per tooth (i.e., mesio-buccal [mb], mid-buccal [b], disto-buccal [db], mesio-oral [mo], mid-oral [o] and disto-oral [do]) at baseline, 3 and 6 months post-treatment:

- Bleeding on probing (BOP) assessed through visual inspection 20 s after probing using a dichotomous scale (present/absent)
- Probing depth (PD) measured in mm from the gingival margin to the bottom of the probed pocket
- Recession (REC) measured in mm from the gingival margin to the cemento-enamel junction or to the margin of a cervical restauration
- Clinical attachment level (CAL) calculated by adding PD and REC at each site

Clinical assessments were performed by a calibrated examiner (U.M.D.) who was not aware of the procedure to be performed. Before the beginning of the study, five patients not involved in the study, each diagnosed with periodontitis stages II–III,^20^ were used to calibrate the examiner. The examiner was asked to evaluate PD, REC, CAL and BOP at 6 sites per tooth at 2 separate appointments, 48 h apart. Calibration was accepted if measurements at baseline and at 48 h were equal to the nearest mm at the >90% level.

### Statistical Analysis

Statistical analysis was performed using IBM SPSS Statistics 27 software (IBM; Armonk, NY, USA). The primary outcome variable was the reduction of PD. The Shapiro-Wilk test was performed to assess whether clinical periodontal measures followed a normal distribution. Statistical analysis was based on the Wilcoxon signed-rank test to assess pre- and post-treatment comparisons. Statistical significance was set at p < 0.05.

## Results

This case series comprised a total of 21 healthy non-smoking patients, 15 females (71.4%) and 6 males (28.6%). The age of the included patients ranged from 33 to 75 years old, with a median age 50 years.

Descriptive statistics for PD, CAL, and BOP at baseline of the study are summarised in Table 1.

**Table 1:** Descriptive characteristics of sample population at the baseline

Patients (n)	21
Median age (range)	50 (33–75)
Gender, n (%)	
Males Females	6 (28.6) 15 (71.4)
PD (mm) Mean ± SD	4.7 ± 0.2
CAL (mm) Mean ± SD	4.9 ± 0.5
BOP (%) Mean ± SD	83.2 ± 15.6

Compared to baseline, a statistically significant mean reduction of PD was obtained after 3 and 6 months, amounting to 2.6 ± 0.4 mm and 2.9 ± 0.4 mm, respectively (p < 0.001).

The difference in PD reduction between the 3- and 6-month follow-ups was 0.3 ± 0.3 mm, and was statistically significant (p = 0.004). Compared to baseline, mean CAL gain amounted to 2.3 ± 0.5 mm at 3 months, and 2.6 ± 0.5 mm at 6 months (p < 0.001). A statistically significant difference of CAL gain was measured between the 3- and 6-month follow-up visits (0.4 ± 0.4 mm, p = 0.016).

A statistically significant reduction of mean BOP values was noted after 3 and 6 months following treatment. In particular, a mean reduction of BOP values compared to baseline reached 54.9 ± 16.9 % at 3 months and 65.6 ± 16.4 % at 6 months (p < 0.001). The BOP reduction between 3- and 6-month follow-ups amounted to 10.7 ± 11.9 % (p < 0.001).

Means (SD) of the differences vs baseline for PD, CAL and BOP are depicted in Table 2.

**Table 2:** Means (SD) of the differences (∆) from baseline for probing depth (PD), clinical attachment level (CAL) and bleeding on probing (BOP)

	Month 3	Month 6	∆ 3 to 6 months
∆ PD (mm)	2.6 (0.4)	2.9 (0.4)	0.3 (0.4)
∆ CAL (mm)	2.3 (0.5)	2.6 (0.5)	0.4 (0.4)
∆ BOP (%)	54.9 (16.9)	65.6(16.4)	10.7 (11.9)

Statistically significant (p < 0.001).

Furthermore, the frequency distribution of shallow (1-3 mm), medium (4-5 mm) and deep (> 6 mm) pockets at baseline, at 3 and at 6 months was analysed (Table 3). At baseline, study subjects exhibited 1803 sites with PD 4-5 mm, which decreased to 414 and 274 sites at the 3- and 6-month follow-ups, respectively. The number of sites ≥ 6 mm decreased from 319 at baseline to 9 at 3 months and to 3 at 6 months.

**Table 3:** Number of sites with shallow (1–3 mm), medium (4–5 mm) and deep (> 6 mm) pockets at different timepoints

	1–3 mm	4–5 mm	≥ 6 mm
Baseline	1603	1803	319
After 3 months	3224	414	9
After 6 months	3375	274	3

## Discussion

The present prospective case-series study investigated the clinical outcomes obtained with subgingival application of sodium hypochlorite/amino acid and a mixture of natural and cross-linked hyaluronic acid (high molecular) gels in conjunction with non-surgical periodontal therapy.

The findings suggest that the adjunctive application of a combination of sodium hypochlorite/amino acid and a mixture of natural and cross-linked hyaluronic acid (high molecular) gels to scaling and root planing in pockets exhibiting a PD ≥ 4 mm led to statistically significant improvements of the investigated clinical parameters. In particular, at 6 months after treatment, the mean PD reduction was 2.9 ± 0.4 mm (p < 0.001), while the mean CAL gain measured 2.6 ± 0.5 mm (p < 0.001). The mean BOP decreased from 83.2 ± 15.5% at baseline to 17.6 ± 11.5 % at the 6-month follow-up (p < 0.001). Interestingly, a statistically significant improvement in PD, CAL and BOP values was observed from the 3- to the 6-month follow-up, even though no further treatment was performed.

An important finding of the present study is the change in the total number of moderate (4-5 mm) and deep (≥ 6 mm) pockets. In particular, the total number of pockets of 4-5 mm was reduced from 1803 to 274 with the corresponding values of 319 and 3 in the deep-pocket (≥ 6 mm) category. As the ultimate goal of non-surgical periodontal therapy is to reduce/eliminate all sites > 4 mm, the ‘Clean and Seal’ technique seemed to be efficient in reducing further treatment need for the residual periodontal pockets.

Another important finding was the uneventful healing of soft tissues; none of the patients reported any adverse reactions or discomfort following therapy. These findings are important, since to the best of our knowledge, this is the first clinical study evaluating the effectiveness of sodium hypochlorite/amino acid and a mixture of natural and cross-linked hyaluronic acid (high molecular) gels in conjunction to SRP in patients with untreated periodontitis.

Justification for the adjunctive application of hyaluronic acid has been provided by several clinical studies.^7,12,21^ Previous clinical data pointed to statistically significant reductions in PD^7,12,21^ and BOP,^12,21^ as well as CAL gains,^21^ following adjunctive hyaluronic acid applications compared to subgingival debridement alone. In line with this, findings of one recent systematic review on non-surgical periodontal therapy pointed to a statistically significant reduction in PD (weighted mean difference (WMD): 0.36 mm; 95%CI: 0.54 to -0.19 mm; p<0.0001), BOP values (-15%; 95%CI: -22 to -8%; p<0.0001) and CAL gain (0.73 mm; 95% CI: 0.28 to 1.17 mm; p<0.0001) following adjunctive topical application of hyaluronic acid over SRP alone.^8^

Clinical effectiveness of the adjunctive use of sodium hypochlorite gel has been evaluated in several clinical studies reporting on non-surgical treatment of residual periodontal pockets (PD ≥5 mm),^17^ non-surgical peri-implant mucositis^9^ and peri-implantitis therapy.^23^ In particular, while treating residual periodontal pockets, greater PD reduction in initially deep residual pockets (≥7 mm) was observed in the adjunctive sodium-hypochlorite gel group. Furthermore, following treatment, only one residual pocket of ≥ 7 mm was still detectable in a test group, whereas six compromised sites persisted in the control group.^17^ Regarding non-surgical peri-mucositis therapy, the adjunctive application of sodium hypochlorite gel led to slightly better PD reduction compared to the control (i.e., mechanical debridement) – from 3.93 ± 1.09 mm to 3.04 ± 0.46 mm (p = 0.0001) and from 3.68 ± 0.85 mm to 3.07 ± 0.58 mm (p = 0.0001), respectively. However, no statistically significant difference was observed between the groups (p=0.53).^9^ Similar results were observed when adding sodium hypochlorite gel adjunctively in non-surgical peri-implantitis therapy.^23^ In particular, the reduction of BOP-positive sites in the test group changed from 0.97 (SD ± 0.12) to 0.38 (SD ± 0.46), and in the control group from 0.97 (SD ± 0.12) to 0.31 (SD ± 0.42), but there were no statistically significant differences between the study groups.

Despite the fact that no statistically significant improvements in PD and BOP could be obtained in the studies mentioned above,^9,17,23^ the test groups always showed a tendency for greater clinical improvements than the controls (i.e., mechanical debridement), thus pointing to the beneficial effect of the adjunctive application of sodium hypochlorite.

When interpreting the results, it must be pointed out that the present case series only provides data from 21 consecutively treated patients without a control group (i.e., SD alone) and with a relatively short follow-up period (i.e., 3 and 6 months). However, despite these limitations, the excellent clinical outcomes coupled with the uneventful healing seem to suggest that this novel treatment concept may be of clinical relevance, thus warranting further investigations. Obviously, randomised controlled clinical trials are needed to validate this treatment concept for non-surgical periodontal therapy.

## Conclusion

Within its limitations, the present case series has shown that a combination of sodium hypochlorite/amino acid and a mixture of natural and cross-linked hyaluronic acid (high molecular) adjunctive to subgingival mechanical debridement may represent a valuable approach to improve the outcomes of non-surgical periodontal treatment.
